# Bioaccessibility of Antimony in Solid Environmental Matrices

**DOI:** 10.3390/toxics14100853

**Published:** 2026-09-24

**Authors:** Valérie Cappuyns, Gitte Ruysschaert

**Affiliations:** Centre for Economics and Corporate Sustainability (CEDON), KU Leuven, Warmoesberg 26, 1000 Brussels, Belgium

**Keywords:** antimony, bioaccessibility, ingestion, human health risk assessment

## Abstract

Antimony (Sb) is a critical raw material whose extraction, processing, and use contribute to environmental contamination and to its enrichment in soils, dusts, slags, and mining residues. Human exposure to Sb occurs through, among others, incidental ingestion of contaminated particles, making oral bioaccessibility an important parameter for human health risk assessment. This study investigated the in vitro oral bioaccessibility of Sb in solid environmental matrices and evaluated the influence of geochemical properties, environmental matrix type, and analytical methodology. A meta-analysis was conducted using data from 20 peer-reviewed studies published between 2009 and 2025, comprising 175 samples of soils, dusts, and mining waste analyzed using established in vitro bioaccessibility methods. Total Sb concentration was identified as the strongest predictor of both gastric and intestinal Sb bioaccessibility. Differences in gastric and intestinal bioaccessibility were observed between soils, dust and mining waste samples, while no conclusions could be drawn for intestinal bioaccessibility. The results demonstrate that Sb bioaccessibility is governed primarily by total Sb concentration, while methodological and mineralogical factors contribute to additional variability. Relative gastric bioaccessibility was also influenced by the in vitro method used, with Method 1340 and the UBM generally yielding lower values than the PBET, SBET, and SBRC. However, without a paired design, any causal conclusion about the optimality of a particular test is premature.

## 1. Introduction

Antimony (Sb) is a critical raw material, indispensable for green energy and high-tech industries. It is mainly extracted through conventional mining, with a few countries controlling nearly 90% of mine production. Antimony mining puts severe pressure on surrounding ecosystems through several factors, including the release of metal(loid)s into the environment. While mining is a significant contributor to environmental Sb contamination, other anthropogenic and natural processes release Sb into the air, soil, and water. Anthropogenic sources include the wear of automotive brake pads [1], and the use of flame retardant in electronics, plastics, and textiles [2]. It is also released through the combustion of fossil fuels and municipal waste [3], and via the weathering of ammunition at shooting ranges [4]. Human exposure to Sb and other metal(loid)s (via dust, water, food, etc.) can result in acute and long-term health effects.

Human biomonitoring and in vivo bioavailability testing using living organisms provide a direct way of assessing the effects of ingesting contaminated particles. In human biomonitoring, however, there are many other environmental factors that influence the accumulation of contaminants and their effects on human health. Moreover, in vivo bioavailability tests raise ethical concerns. For these reasons, in vitro bioaccessibility tests have been developed which do not use living organisms but simulate the conditions in the gastrointestinal tract in the laboratory. In vitro bioaccessibility tests replicate the chemical and physiological environment of the human digestive tract using one or more distinct, highly controlled sequential phases: the oral or saliva phase mimics the conditions in the mouth using synthetic saliva and neutral pH (pH 6.5–7.0); the gastric phase mimics the stomach (low pH (usually pH 1.5–2.5) and digestive enzymes, such as pepsin or glycine); and the intestinal phase mimics the small intestine (neutral pH ~6.5–7.0 and bile salts and pancreatin enzymes). In vitro bioaccessibility refers to the amount of a substance released from a matrix into the gastrointestinal environment under in vitro conditions. This does not automatically equate to the amount subsequently absorbed by the system, but is considered a reasonable approximation. Table 1 provides an overview of commonly used in vitro bioaccessibility tests that are also addressed in the present study; a table with more details on the operational parameters can be found in the Supplementary Materials (Table S1). The SBET (Simplified Bioaccessibility Extraction Test) and Method 1340 include only a stomach compartment (gastric phase) (Table 1), whilst the PBET (Physiologically Based Extraction Test), UBM (Unified BARGE Method) and SBRC (Solubility/Bioavailability Research Consortium) cover both the stomach and intestinal compartments. The saliva phase is only included in the UBM.

Bioaccessibility in soil, dust, waste materials, etc., is well studied for many elements like Pb, As, and Cd [5,6], but there is far less information available on the bioaccessibility of Sb in solid environmental matrices [6]. The aim of the present study was to investigate the in vitro bioaccessibility of Sb in mining waste, dust, and soils, in relation to the geochemical and mineralogical properties of these matrices. Therefore, a meta-analysis of geochemical data (including oral bioaccessibility) and mineralogical data from dust, mining residues and soil samples published in the peer-reviewed literature was performed to evaluate the influence of geochemical properties, environmental matrix type, and test methodology. To improve comparability between different tests, results of Sb bioaccessibility in the gastric and intestinal phase will be addressed separately, and the saliva phase will not be considered in the present paper.

**Table 1:** Overview of in vitro bioaccessibility tests addressed in the present work.

Name	Abbreviation	Phases	Reference
Physiologically Based Extraction Test	PBET	Gastric + intestinal	[7]
Simplified Bioaccessibility Extraction Test	SBET	Gastric	[8]
Solubility/Bioavailability Research Consortium	SBRC	Gastric (SBRC I) + intestinal (SBRC II)	[9,10]
Unified BARGE Method	UBM	Saliva + gastric + intestinal	[11]
Test Method 1340: In Vitro Bioaccessibility Assay for Lead in Soil	Method 1340	Gastric	[12]

## 2. Materials and Methods

### 2.1. Data Collection

Literature searches were conducted in different databases (Web of Science, Elsevier Science Direct, PubMed, and Springer Link) using a combination of Boolean operators and keywords, including (“antimony” OR “Sb”) AND (“bioavailability” OR “bioaccessibility”) AND “in-vitro” AND (“soil” OR “waste” OR “dust” OR “PM” OR “particulate matter”). To ensure comprehensive coverage, additional literature was identified through backward and forward citation tracking (snowballing). After removing duplicates, the remaining articles underwent a sequential screening process based on title, abstract, and full-text assessment against specific eligibility criteria.

Studies were included if they evaluated oral human health-related bioaccessibility of Sb using established in vitro methodologies (e.g., the PBET, SBET, SBRC, UBM, or US EPA Method 1340) and environmental matrices such as soil, dust, and mining residues. Publications were excluded if they did not provide data on (in vitro) Sb bioaccessibility, or focused on ecological receptors such as plants or crops, or examined pure chemical compounds (e.g., Sb_2_S_3_, Sb_2_O_3_ or C_8_H_4_K_2_OSb_2_ [13]). The temporal scope was restricted to articles published from 2009, when Denys et al. [14] published the first in vitro study dedicated specifically to the bioaccessibility and speciation of Sb in soils, until December 2025.

Data from the selected articles were extracted and compiled into a structured database (Microsoft Excel, Office 365), where each row represented an individual sample. Consequently, publications evaluating multiple samples contributed multiple entries to the dataset. Information was systematically retrieved from the main text, methodologies, and results sections of each article. The extracted data were classified into three main categories:

- Bibliographic information: Author name and publication year.
- Contextual information: Type of in vitro assay, geographic location, sample type, and source of antimony contamination.
- Sample properties: Sample identifier/code, pH, total concentration of Sb in mg/kg, gastric and intestinal bioaccessibility of Sb in mg/kg and/or %, organic carbon in mg/kg, total carbonate in g/kg, nitrogen in g/kg, exchangeable phosphorus in mg/kg, soil, cation exchange capacity in cmol/kg, the texture of the soils, clay content, silty sand content, sand content and organic matter in mg/kg. When data were missing from the text or Supplementary Materials and only available graphically, values were digitized and extracted from figures using PlotDigitizer https://plotdigitizer.com/ (accessed on 3 March 2026).

To ensure data integrity and compatibility for statistical analysis, the raw dataset—comprising heterogeneous measurements derived from diverse studies, sample types, and analytical protocols—was initially harmonized within a Microsoft Excel environment. Subsequent data processing was executed using IBM SPSS Statistics Version 31, which was utilized for data cleaning, variable transformation and recoding, verification, diagnostic testing, and exploratory data visualization.

### 2.2. Data Cleanup

The dataset was imported into IBM SPSS Statistics, where all variables were systematically evaluated for completeness, consistency, and measurement level (nominal, ordinal, or scale). Variables representing organic matter (OM), soil organic matter (SOM), and organic carbon (OC) were harmonized, using a conversion factor of 1.9 for OC [15]. Variable types were verified to ensure numerical attributes were correctly defined as scale variables, while categorical attributes, such as the sample type and in vitro method, were designated as nominal variables.

During this verification phase, data discrepancies were rectified; specifically, several numerical variables that had been improperly classified as character strings due to embedded spaces, formatting irregularities, or notations for detection limit (e.g., ‘<DL’) were corrected. These entries were processed using string-to-numeric conversion functions, ensuring complete data retention during the recoding workflow.

### 2.3. Statistical Data Analysis

To assess the appropriateness of combining different studies a heterogeneity test was applied. For each variable (total Sb, Fe, Al, and Mn concentration, pH, organic matter) the correlation coefficients with gastric and intestinal Sb bioaccessibility were calculated on a per article basis. These coefficients were then converted into Fisher’s Z-values using Fisher’s Z-transformation [16]. Heterogeneity was evaluated using Higgins’ I^2^ statistic, which is a widely recommended measure for quantifying heterogeneity in meta-analyses [17]. To account for potential non-independence among samples originating from the same publication, a linear mixed-effects model including publication as a random intercept was fitted.

For each variable the descriptive statistics were calculated, including the minimum, maximum, mean, and standard deviation. The distribution of the data was right-skewed, and therefore a log10 transformation was applied to improve normality and stabilize the variance [18]. Since the transformed variables remained slightly skewed, the regression models were interpreted with caution. Residual diagnostics were performed to ensure that the model assumptions were adequately met [19]. Two-tailed Pearson correlation coefficients were calculated for the original and log10-transformed values to investigate the linear relationships between different variables.

A stepwise multiple linear regression analysis was performed to investigate the relationship between bioaccessible Sb as the dependent variable and two or more independent variables. The dependent variables were gastric or intestinal bioaccessibility of Sb and the independent variables were pH, total concentration of Sb, Fe, and Mn, and organic matter content. The regression was performed using the stepwise method with backward elimination in order to include the most significant independent variables. The multiple linear regression assumptions were tested by creating a scatter plot of residuals versus predicted values to confirm homoscedasticity. To check for the presence of multicollinearity, the Variance Inflation Factor (VIF) was calculated. Following the common rule of thumb established by Kim [20], a VIF value of 5 or higher was considered an indication of problematic multicollinearity.

A one-way ANOVA was employed to evaluate differences in Sb bioaccessibility across various environmental matrices and in vitro methods. To account for unequal variances and sample sizes among groups, Welch’s ANOVA was utilized. Statistically significant differences identified by Welch’s ANOVA were subsequently isolated using the Games-Howell post hoc test for pairwise comparisons [21].

## 3. Results

### 3.1. Literature Data on In Vitro Bioaccessibility of Sb

The schematic overview of the literature screening process, presented in a PRISMA diagram, is presented in the Supplementary Materials (Figure S1). Using the search terms and criteria that were described earlier, a total of 268 articles were selected and added to Zotero. After removing duplicates, 253 articles were screened, based on the title, which excluded 127 articles. Screening of the abstracts resulted in the additional exclusion of 45 articles. Full-text reading revealed that several articles investigated elements other than Sb (39), or did not address in vitro Sb bioaccessibility (18). Studies addressing plant or animal uptake of antimony were also excluded. Finally, the dataset contained data on 175 samples collected from 20 articles (Table 2).

With respect to the type of samples investigated in the different papers, most samples (96) were classified as ‘soil’, including agricultural soil and soil from residential areas, soil contaminated by mining activities or municipal waste. The ‘dust’ category (62 samples) was defined as fine particulate matter originating from mechanical disturbance or weathering of soils, consisting of mineral particles reflecting the composition of the parent material [41]. Dust is sometimes described based on its origin, such as household dust and street dust, or based on its grainsize (e.g., PM_10_). The definition of mining residue (22 samples) is taken from EU Directive 2006/21/EC, which refers to waste generated during the exploration, extraction, processing and storage of minerals and during the exploration of mines. This category includes mining-related materials such as mine dust, mine tailing dust, tailings, mine waste and certain forms of waste rock or mined bedrock. Mining dust is classified under the category of mining residue rather than dust, as its mineralogical properties are more consistent with the former.

The samples originate from all over the world. The majority (41%) of samples were subjected to the in vitro PBET (72 samples). The second most applied test was the SBRC (23% of the samples in the dataset), followed by the UBM (17.7% of the samples). The SBET and Method 1340 were applied in four and three articles respectively.

Table 3 shows the minimum, maximum, mean and standard deviation of gastric and intestinal bioaccessibility of Sb, total concentration of Sb, organic matter (OM), Fe, Al and Mn.

With respect to heterogeneity, the results of the Higgins test are presented in Table S2 (Supplementary Materials) and indicate the following: moderate heterogeneity for total Sb concentration, pH, Fe, and Mn, and high but acceptable heterogeneity for Al regarding gastric Sb bioaccessibility. Due to excessive heterogeneity (I^2^ > 85%) for Al with respect to intestinal bioaccessibility, this was excluded for further analysis. A linear mixed-effects model including the publication as a random intercept gave results that were qualitatively identical to those obtained from the linear regression, indicating that study-level clustering did not materially affect the conclusions.

From the descriptive statistics (Table 3), we can see that most variables are right-skewed, characterized by very high maximum values, low minimum values and standard deviations that exceed the mean. This skewed distribution has also been confirmed by generating normal distributions, which exhibited the same pattern. This pattern is typical of environmental data [42,43] and confirms the need for data log10 transformation for further analysis. This transformation was necessary to obtain normally distributed data for gastric and intestinal bioaccessible Sb, organic matter, and total concentrations of Fe, and Mn. Figure 1 shows the relationship between the total Sb concentration (mg/kg, on a logarithmic scale) and the gastric and intestinal Sb bioaccessibility (mg/kg).

### 3.2. Effect of Sample Properties and Matrix Type on Bioaccessibility

The Pearson correlation matrix of the data is included in the Supplementary Materials (Table S3). From Figure 1, the significant positive correlation between log(Sb_TOTAL_) and log(Sb_G_) (r = 0.832, *p* < 0.01) and log(Sb_TOT_) and log(Sb_IN_) (r = 0.881, *p* < 0.01) could already be deduced. Positive correlations were also observed between log(Sb_G_) and log(Fe) (r = 0.341, *p* < 0.01), as well as a negative correlation with organic matter (log(OM)) content (r = −0.342, *p* < 0.05). Log-transformed total concentrations of Fe and Mn also show a strong and significant correlation (r = 0.971, *p* < 0.01), which might indicate a shared geochemical compound. Similarly, the pH correlates positively with log(Fe) (r = 0.552, *p* < 0.01).

The relationships between Sb bioaccessibility and geochemical variables were evaluated using multiple linear regression. Separate models were developed for gastric and intestinal Sb bioaccessibility. When the complete dataset was considered, a linear relationship was found between log(Sb_IN_) and log(Sb_TOT_), as well as a linear relationship between log(Sb_G_) and (log(Sb_TOT_) and log(Fe)).

The linearity of the log(Sb_G_) or log(Sb_IN_) versus log(Sb_TOT_) relationship was evaluated by including a quadratic term [log(Sb_TOT_)]^2^ in the model. For gastric bioaccessibility, the quadratic term [log(Sb_TOT_)]^2^ was not significant (*p* = 0.846), indicating no evidence of departure from linearity. In addition, a Ramsey RESET test based on squared and cubic fitted values was performed. The Ramsey RESET test was significant (*p* < 0.001), suggesting potential model misspecification due to omitted variables, interactions, or more complex nonlinear effects. Therefore, the different matrices were investigated separately (Table S4).

For soil and mine waste, only total Sb concentrations (log(Sb_TOT_)) appeared to be a significant variable to explain gastric Sb bioaccessibility (log(Sb_G_)). The quadratic term was not significant (*p* = 0.745 and 0.428 for soil and mine waste, respectively), indicating no evidence of departure from linearity. For dust, the squared term [log(Sb_TOT_)]^2^ was the only significant variable after including it in the regression together with log(Sb_TOT_), pointing to departure from linearity (Table 4).

With respect to intestinal bioaccessibility of Sb, a linear relationship between total and bioaccessible Sb concentrations could only be established for soil samples.

### 3.3. Effect of In Vitro Test on Bioaccessibility

The in vitro methods differ from each other in a number of respects: the amount of sample used in the test (0.4 g for the PBET, SBET and SBRC to 1 g for Method 1340), and the grainsize of the samples, with most methods using a grainsize < 250 µm, and Method 1340 using samples with grainsizes < 150 μm. The SRBC method prescribes a suspension mixer to stir the mixture, operating at 40 rotations per minute (rpm). The other methods use end-over-end rotation at speeds ranging from 30 rpm to 60 rpm. The temperature, incubation time and filtration are the same for most in vitro methods, while the centrifugation conditions often differ (Table S1, Supplementary Materials).

To statistically test whether the in vitro bioaccessibility of Sb is determined by different in vitro methods, a one-way ANOVA and Games–Howell post hoc test were performed (Table 5).

According to Welch’s ANOVA test, there are significant differences between the various in vitro methods for (relative) gastric bioaccessibility (*p* < 0.001). The Games–Howell post hoc analysis indicates that the in vitro PBET, SBET and SBRC methods do not differ significantly from one another; they therefore yield comparable relative gastric bioaccessibility of Sb. All other bioaccessibility tests differed significantly from these three tests with a *p*-value < 0.001. The results of Welch’s ANOVA test for intestinal bioaccessibility (Table 5) again demonstrated that the different in vitro methods differ significantly from each other (*p* = 0.002). From the Games–Howell post hoc analysis, we can conclude that, in terms of intestinal bioaccessibility of Sb, the PBET differs significantly from the SBRC and the SBRC is significantly different from the UBM (*p* < 0.05). A definitive conclusion is, however, precluded by the lack of a paired design comparing identical samples across multiple analytical methods.

## 4. Discussion

### 4.1. Controlling Factors for Sb In Vitro Bioaccessibility

A significant positive correlation was observed between total (log-transformed) Sb concentration and both gastric and intestinal bioaccessibility. This finding is consistent with previous studies that reported positive associations between total Sb concentrations and bioaccessibility [22,34,38]. However, contrasting results have also been reported in the literature. For example, Kastury et al. [39] found no significant relationship between total Sb concentration and bioaccessibility, suggesting that factors other than total concentration may influence Sb bioaccessibility.

pH was not retained as a variable to explain gastric and intestinal bioaccessibility of Sb. In soil, pH is widely recognized as a key factor controlling the mobility, speciation, and thus also the bioaccessibility of antimony (Sb) in soils. Increasing pH often decreases Sb adsorption due to competition with hydroxide ions for sorption sites and the increasingly negative surface charge of soil minerals, thereby enhancing Sb mobility [35,44]. Acidic soils tend to exhibit lower Sb mobility and bioaccessibility than alkaline soils, due to stronger interactions of Sb with mineral surfaces at low pH [35]. Furthermore, pH impacts Sb speciation, with Sb(V) being the dominant form under oxidizing conditions. Sb(V) is generally more mobile than Sb(III) because it occurs mainly as the anionic species Sb(OH)_6_^−^ [44]. Consequently, variations in soil pH (as well as redox conditions) can significantly affect the fraction of Sb that becomes bioaccessible in the gastrointestinal environment, making pH an important parameter to consider when assessing human health risks associated with Sb-contaminated soils. In mine waste and contaminated dust, pH is also a critical factor controlling Sb dissolution and bioaccessibility. In Sb-contaminated mining residues and metallurgical dust, changes in pH substantially altered the fraction of Sb available for gastrointestinal solubilization [4,35].

Under acidic conditions, Sb is generally more strongly retained by Fe, Al, and Mn oxy(hydr)oxides, which can reduce its mobility and bioaccessibility. Therefore, Fe and Mn were initially included in the regression analysis because of their well-documented influence on Sb mobility in soils [35,44]. Li et al. [26] reported negative correlations between Fe, Al, and Mn concentrations and Sb bioaccessibility. In the present study, however, Fe and Mn were not retained in the model explaining gastric and intestinal Sb bioaccessibility, except for Fe, which became a significant predictor for Sb gastric bioaccessibility in soil in a model with log(Sb_TOT_), and log(Fe) as independent variables. Including a quadratic term for Fe (log(Fe))^2^ in the regression showed that this term was significant, suggesting model misspecification. Moreover, due to many missing values, the sample size was reduced to 88, which is around 50% of the original dataset for soils.

Antimony in mining residues commonly occurs in primary sulfide minerals such as stibnite (Sb_2_S_3_), or is adsorbed onto secondary Fe(hydr)oxides formed during weathering. Under acidic conditions, the dissolution of Sb-bearing minerals and associated metal oxides can increase, promoting the release of Sb into solution and enhancing its bioaccessibility. In mining wastes, mineralogy (stibnite, pyrite scorodite, Fe-oxides, jarosite, etc.) most likely has a significant influence on Sb bioaccessibility [45]. Several studies on mine wastes and smelter-derived dusts have shown that Sb exhibits elevated bioaccessibility in simulated gastric fluids due to the low pH of the extraction medium, which enhances mineral dissolution and desorption processes. In contrast, bioaccessibility generally decreases under near-neutral intestinal conditions as a result of Sb precipitation and re-adsorption onto mineral surfaces [4,35].

### 4.2. Methodological Differences

Differences in Sb bioaccessibility reported among different studies may partly reflect methodological differences between in vitro extraction procedures. Extraction parameters (liquid: solid ratio, time, rotation speed, temperature, etc.) differ between the methods (Table S1), but are included in the model only nominally.

Because Sb dissolution is highly sensitive to extraction pH, solution chemistry, L/S ratio, etc., different bioaccessibility tests can yield substantially different results for the bioaccessible Sb fraction. This is also the case for mining wastes and metallurgical dusts where Sb occurs in a range of primary and secondary mineral phases, with a different reactivity [45]. Denys et al. [14] concluded that differences among in vitro bioaccessibility tests relate to the experimental design, and the most reliable method is the one that has been validated against in vivo bioaccessibility data, such as the UBM for As, Cd, and Pb.

A further point to consider is that comparing the literature data on total element concentrations is also not completely straightforward, since different studies use different methods to determine ‘total’ Sb concentration. The overview in Table S5 shows that different methods are used to determine total concentrations of Sb in soils, dust and waste materials. Cappuyns et al. [46] compared different methods to determine (pseudo-)total Sb concentrations in mining waste and soils. LiBO_2_ fusion generally yielded higher and more reliable total Sb concentrations than acid digestion, especially for some mine-waste samples. This indicated that acid digestion did not always completely dissolve Sb-containing mineral phases. This is an additional argument to include (quantitative) mineralogical analyses in bioaccessibility studies of mining waste.

For quality control, the analysis of a certified reference material for total element concentrations has become common practice (Table S5). However, for the in vitro bioaccessibility of Sb, no information about the use of certified reference materials was found. In a few studies [29,30], the accuracy of the analysis of the leachates is assessed with certified reference materials, but this is only informative for the quality of the chemical analysis, not for the bioaccessibility test itself. Developing certified reference materials (soils, dust, etc.) for in vitro bioaccessibility tests would be an important step forward.

### 4.3. Use in Human Health Risk Assessment

In vitro oral bioaccessibility tests are increasingly used in human health risk assessment to provide a more realistic estimate of the fraction of a contaminant that can actually dissolve in the gastrointestinal tract and become available for absorption after ingestion. Rather than assuming that 100% of a contaminant present in soil, dust, or other environmental media is absorbed, bioaccessibility testing helps refine exposure estimates and reduce uncertainties [47].

Using total concentrations results in a very conservative prediction, most likely an overestimation, of human health risk. For example, He et al. [48] calculated human health risks of Sb in a mining area based on total concentrations (Sb_total_ = 1.19–2155.44 mg/kg). The present study shows that the bioaccessibility of Sb in soil, dust and mining waste is relatively low, pointing to the importance of bioaccessibility values for human health risk assessments.

## 5. Conclusions

This study provides a comprehensive assessment of the in vitro oral bioaccessibility of antimony (Sb) in solid environmental matrices contaminated by mining and other anthropogenic activities. By integrating data from 20 published studies, including 175 individual samples, the meta-analysis allowed the identification of key factors controlling Sb bioaccessibility and the evaluation of methodological influences on reported results.

The results demonstrate that total Sb concentration is the strongest and most consistent predictor of both gastric and intestinal bioaccessibility. Significant positive relationships were observed between total Sb concentrations and bioaccessible Sb, indicating that highly contaminated materials generally release larger amounts of Sb under simulated gastrointestinal conditions. pH was not retained as a significant factor explaining Sb gastric and intestinal bioaccessibility. Despite the well-established influence of pH on Sb speciation, sorption, and mobility, it is also linked to the mineralogical environment, redox conditions, etc., and can thus not be interpreted in isolation. Total Fe concentrations showed limited predictive power when examined together with total Sb, despite the recognized role of Fe-containing compounds in controlling Sb retention and release in the environment.

The type of environmental matrix had an influence on gastric Sb bioaccessibility, suggesting that broad matrix classifications are important when evaluating Sb exposure risks. However, considerable variability remained unexplained, highlighting the potential influence of geochemical and mineralogical factors. The occurrence of Sb in different primary and secondary mineral phases, including stibnite and associations with Fe-oxyhydroxides, is likely to have a major influence on Sb dissolution and deserves greater attention in future studies.

Methodological differences among in vitro assays significantly influenced bioaccessibility estimates. The PBET, SBET and SBRC generally yielded comparable gastric results, whereas the UBM and Method 1340 produced significantly lower values. These discrepancies emphasize the need for greater harmonization of procedures and pairwise testing to clarify differences between in vitro methods. Validating Sb bioaccessibility testing remains essential, particularly through comparisons with in vivo bioavailability data.

Overall, the findings confirm that the use of total Sb concentrations alone may substantially overestimate potential human exposure. Incorporating bioaccessibility measurements into human health risk assessments can provide more realistic exposure estimates and improve risk-management decisions for Sb-contaminated soils, dusts, and mining wastes. Future research should therefore focus on linking Sb mineralogy and speciation to bioaccessibility, and on establishing one simple, standardized, and validated method for Sb bioaccessibility assessment, including certified reference materials.

## Figures and Tables

**Figure 1:**
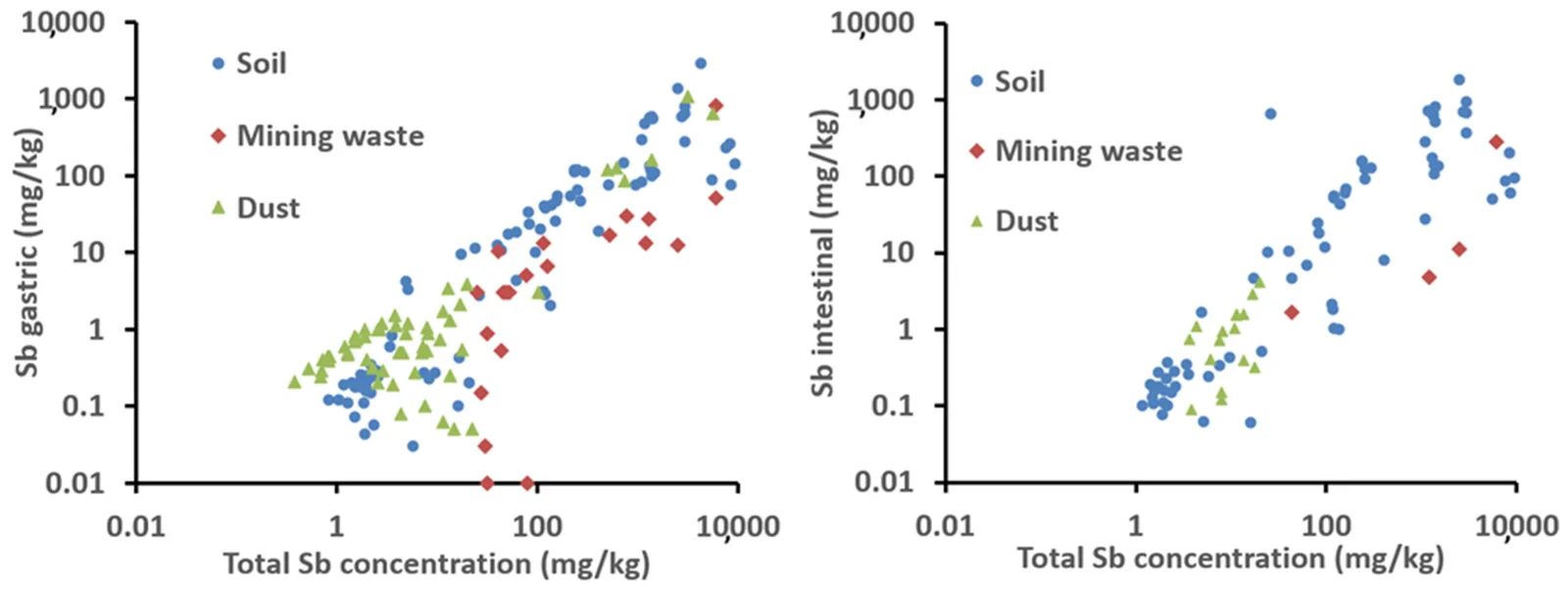
Scatterplot of gastric (left) and intestinal bioaccessibility (right) as a function of total Sb concentration.

**Table 2:** Articles from which data were collected for the meta-analysis, *n* = number of samples.

	Year	*n*	In Vitro Method	Location	Sample Type
[14]	2009	4	UBM	France	Soil, slag & mined bedrock
[22]	2012	4	SBET	Australia	Soil
[23]	2014	2	UBM	Scotland	Soil
[24]	2014	1	SBET	Iran	Soil
[25]	2014	3	SBET	Wales	Soil, swamp & levee
[26]	2014	6	PBET	China	Soil, waste rock & tailings
[27]	2018	5	UBM	Portugal	Dust
[28]	2018	1	SBRC	China	Dust
[29]	2018	9	Method 1340	Brazil	Slag & dust
[30]	2019	17	SBRC	Namibia	Mine tailing dust, slag dust & smelter dust
[31]	2019	28	PBET	Spain	PM_10_
[32]	2019	3	SBRC	Sardinia	Soil
[33]	2020	15	PBET	Ghana	Soil
[34]	2021	3	PBET	China	Soil
[35]	2021	20	SBRC	Sardinia	Soil
[36]	2022	1	Method 1340	Ghana	Soil
[37]	2022	20	PBET	Slovakia	Soil
[38]	2023	8	SBET	China	Soil
[39]	2024	5	Method 1340	Australia	Mine waste
[40]	2024	20	UBM	Slovenia	Soil & dust

**Table 3:** Descriptives of the dataset (*n* = number of samples for which data were available).

	***n***	**Minimum**	**Maximum**	**Mean**	**Std. Dev.**
Sb_G_ (mg/kg)	175	0.0006	2880	105.8	326.0
Sb_IN_ (mg/kg)	92	0.008	1815	121.7	277.2
Sb_TOT_ (mg/kg)	173	0.38	16,600	811.2	2246
pH	100	1.80	9.32	6.96	1.30
OM (mg/kg)	41	4380	125,160	35,701	29,512
Fe (mg/kg)	88	17.90	245,000	22,317	45,307
Al (mg/kg)	74	11.40	110,000	14,250	23,511
Mn (mg/kg)	82	0.70	3860	594	835

**Table 4:** Regression equations with gastric (Sb_G_) or intestinal (Sb_IN_) Sb bioaccessibility as dependent variables and total Sb concentrations (Sb_TOT_) as independent variable (*p* < 0.001).

Gastric Bioaccessibility		R^2^
Soil	log(Sb_G_) = −1.158 + 1.050·log(Sb_TOT_)	0.733
Dust	log(Sb_G_) = −0.495 + 0.237·[log(Sb_TOT_)]^2^	0.819
Mine waste	log(Sb_G_) = −2.301 + 1.209·log(Sb_TOT_)	0.709
**Intestinal Bioaccessibility**		
Soil	log(Sb_IN_) = −0.986 + 1.005·log(Sb_TOT_)	0.77

**Table 5:** ANOVA and post hoc test of relative bioaccessibility and in vitro methods.

One-Way ANOVA		Games–Howell	
Relative Bioaccessibility	*p*-Value	Bioaccessibility Test Method	*p*-Value
Sb Gastric	*p* < 0.001	PBET–Method 1340	*p* < 0.001
		PBET-SBET	*p* = 0.007
		PBET-SBRC	*p* = 0.902
		PBET-UBM	*p* < 0.001
		SBET–Method 1340	*p* < 0.001
		SBET-SBRC	*p* = 0.965
		SBET-UBM	*p* = 0.002
		SBRC–Method 1340	*p* < 0.001
		SBRC-UBM	*p* < 0.001
		UBM–Method 1340	*p* = 0.007
Sb Intestinal	*p* = 0.002	PBET-SBET	*p* = 0.935
		PBET-SBRC	*p* < 0.001
		PBET-UBM	*p* = 0.999
		SBET-SBRC	*p* = 0.596
		SBET-UBM	*p* = 0.919
		SBRC-UBM	*p* < 0.001

## Data Availability

The original contributions presented in this study are included in the article/Supplementary Materials. Further inquiries can be directed to the corresponding author.

## Supplementary Materials

The following supporting information can be downloaded at: https://www.mdpi.com/article/10.3390/toxics14100853/s1, Table S1: Operational parameters of the different in vitro bio-accessibility tests assessed in the present study; Table S2: Results of the Higgins test for heterogeneity; Table S3 Two tailed Pearson correlation matrix for log transformed data; Table S4 Output for the stepwise linear regression and quadratic test; Table S5: Overview of sample digestion methods, bioaccessibility tests, analytical techniques, samples characteristics, reference materials; Figure S1 PRISMA flowchart of article selection.
