# Supplementary material for: Bioaccessibility of Antimony in Solid Environmental Matrices
Source: Toxics. 2026 Sep 24;14(10):853. doi: 10.3390/toxics14100853 (PMC13709358; doi:10.3390/toxics14100853)
Supplement: Supplementary file 1 [file toxics-14-00853-s001.zip › toxics-4560713-supplementary.pdf]

Supplementary Materials

Table S1: Operational parameters of the different in vitro bioaccessibility tests assessed in the present study

		PBET	SBET	UBM	SBRC (G & I)	Method 1340
Reference		Ruby et al. (1996)	Ruby et al. (1999)	Wragg et al. (2009)	Kelley et al. (2002)	US EPA (2013)
Operational parameters	Amount of soil added	0.4g	0.4g	0.6g	0.4g	1g
	Soil-to-fluid ratio	1/100	1/100	jan/15	1/100	1/100
	Particle size	<250µm	<250µm	<250µm	<250µm	<150µm
	Temperature	37°C	37°C	37°C	37°C	37°C
	Mixing	End-over-end rotation (60rpm)	End-over-end rotation (30rpm)	End-over-end rotation	Suspension mixer (40rpm)	End-over-end rotation (30rpm)
	Centrifugation	3500g 15min	3500g 15min	4500g 15min	2621g 5min	
	Filtration	0.45µm	0.45µm	0.45µm	0.45µm filter	0.45µm filter
Saliva phase	Fluid composition			KCl, NaH_2PO4, KSCN, Na_2SO_4, NaCl		
	Volume			9 ml		
	pH			6.5 ± 0.5		
	Incubation time			5 minutes		
Stomach phase	Fluid composition	Pepsin, sodium malate, sodium citrate, lactic acid and acetic acid	Glycine	NaCl, NaH_2PO_4, KCl, $CaCl_2$, NH_4Cl, glucose, glucuronic acid, urea, glucosamine hydrochloride, BSA, mucin and HCl	Glycine	Glycine and HCl
	Volume	40ml	50ml	13,5ml	40ml	100ml
	pH	2.5	1.5	1.2	1.5	1.5
	Incubation time	1h	1h	1h	1h	1h
Gastric phase	Fluid composition	Bile and pancreatin		Duodenal: NaCl, NaH_2PO_4, KH_2PO_4, KCl, $MgCl_2$, urea, $CaCl_2$, BSA, pancreatin and lipase<br>Bile: NaCl, $NaHCO_3$, KCl, urea, $CaCl_2$, BSA, HCl and bile	Bile and pancreatin	
	Volume			Duodenal: 27ml<br>Bile: 9 ml		
	pH	7		6,3	7	
	Incubation time	4h		4h	4h	

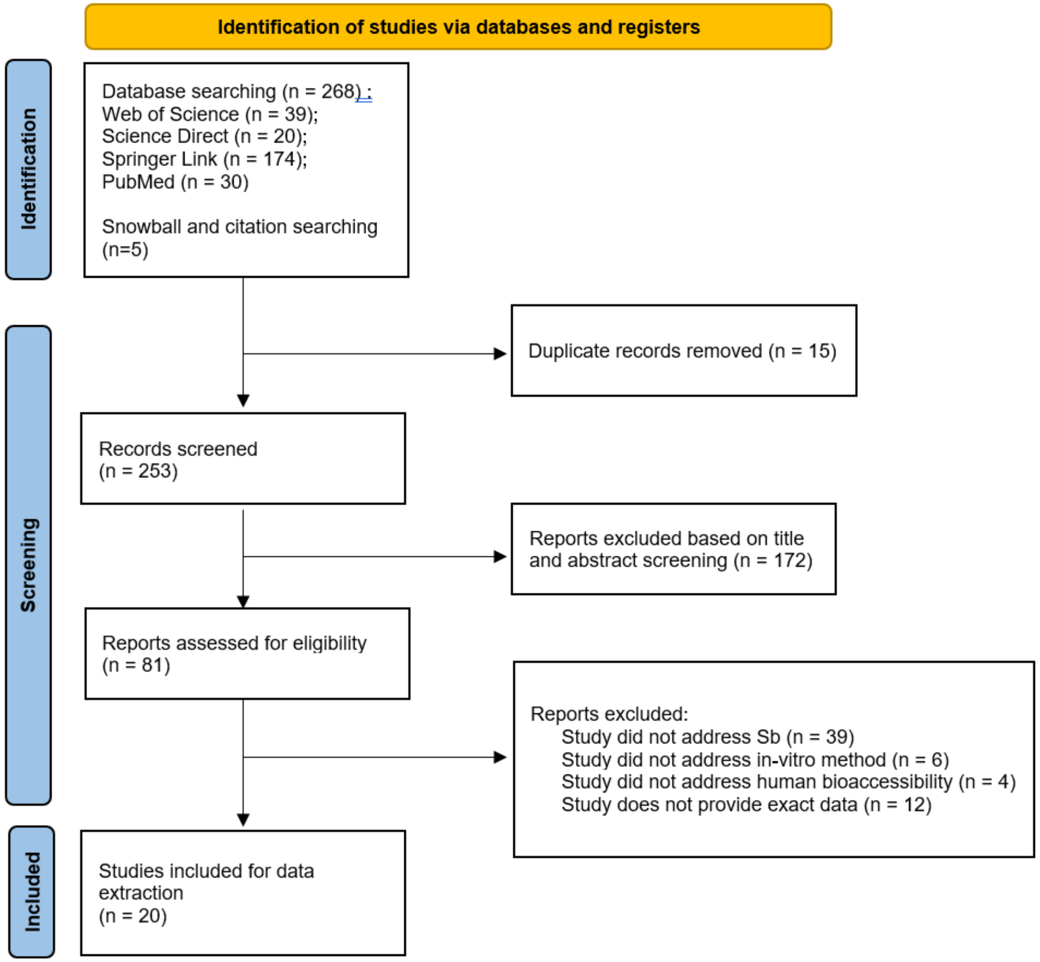

Figure S1 PRISMA flowchart of article selection

Table S2 Results of the Higgins test for heterogeneity

I^2	Sb Gastric Bioaccessibility	Sb Intestinal Bioaccessibility
Total Sb concentration	50.65%	43.84%
pH	58.03%	65.55%
Organic matter (OM)	0%	0%
Fe	64.38%	25.21%
Al	77.4%	88.5%
Mn	49.28%	67.75%

Table S3 Two tailed Pearson correlation matrix for log transformed data

	Sb_G-LOG10	Sb_{IN}-LOG10	Sb_{TOT}-LOG10	pH	OM-LOG10	Fe-LOG10	Al-LOG10	Mn-LOG10
**Sb_G-LOG10**	1							
**Sb_{IN}-LOG10**	0.939**	1						
**Sb_{TOT}-LOG10**	0.832**	0.881**	1					
**pH**	-0.121	-0.071	-0.074	1				
**OM-LOG10**	-0.342*	-0.067	-0.396*	0.017	1			
**Fe-LOG10**	0.341**	-0.222	0.509**	0.552**	0.300	1		
**Al-LOG10**	0.297*	-0.080	0.722**	0.626**	0.013	0.906**	1	
**Mn-LOG10**	-0.041	-0.195	0.477**	-0.172	0.454	0.971**	0.888**	1
	* p<0.05<br>** p<0.01				Sb_G = gastric bioaccessibility of antimony<br>Sb_{IN} = intestinal bioaccessibility of antimony<br>Sb_{TOT} = total concentration of antimony			

Table S4 Output for the stepwise linear regression and quadratic test

Gastric bioaccessibility- soil

Coefficients[a]

Model		Unstandardized Coefficients B	Unstandardized Coefficients Std. Error	Standardized Coefficients Beta	t	Sig.	Collinearity Statistics Tolerance	Collinearity Statistics VIF
1	(Constant)	-16.422	28.249		-.581	.582		
	log10_Sb_Total	1.451	.507	1.202	2.864	.029	.266	3.757
	log10_OM	-.440	.732	-.153	-.601	.570	.722	1.384
	log10_Fe	3.533	5.282	.279	.669	.528	.269	3.722
	log10_Mn	-2.755	2.618	-.293	-1.052	.333	.606	1.651
	pH	1.052	.976	.447	1.078	.323	.272	3.671
2	(Constant)	-17.122	26.908		-.636	.545		
	log10_Sb_Total	1.326	.440	1.098	3.012	.020	.320	3.121
	log10_Fe	3.060	4.980	.242	.614	.558	.275	3.640
	log10_Mn	-2.157	2.309	-.229	-.934	.381	.708	1.413
	pH	.982	.924	.417	1.063	.323	.276	3.619
3	(Constant)	-1.154	6.702		-.172	.868		
	log10_Sb_Total	1.119	.273	.927	4.099	.003	.768	1.302
	log10_Mn	-1.516	1.978	-.161	-.766	.466	.889	1.125
	pH	.515	.505	.219	1.021	.337	.853	1.173
4	(Constant)	-5.087	4.207		-1.209	.257		
	log10_Sb_Total	1.053	.253	.872	4.161	.002	.853	1.172
	pH	.508	.493	.216	1.030	.330	.853	1.172
5	(Constant)	-.806	.649		-1.242	.243		
	log10_Sb_Total	.953	.234	.789	4.066	.002	1.000	1.000

a. Dependent Variable: log10_Sb_Gastric

Coefficients[a]

Model		Unstandardized Coefficients B	Unstandardized Coefficients Std. Error	Standardized Coefficients Beta	t	Sig.	Collinearity Statistics Tolerance	Collinearity Statistics VIF
1	(Constant)	-1.158	.143		-8.117	<.001		
	log10_Sb_Total	1.050	.066	.856	15.910	<.001	1.000	1.000

a. Dependent Variable: log10_Sb_Gastric

Coefficients[a]

Model		Unstandardized Coefficients B	Unstandardized Coefficients Std. Error	Standardized Coefficients Beta	t	Sig.	Collinearity Statistics Tolerance	Collinearity Statistics VIF
1	(Constant)	-1.118	.189		-5.910	<.001		
	log10_Sb_Total	.974	.241	.795	4.052	<.001	.076	13.152
	LogSbTsq	.021	.063	.064	.327	.745	.076	13.152
2	(Constant)	-1.158	.143		-8.117	<.001		
	log10_Sb_Total	1.050	.066	.856	15.910	<.001	1.000	1.000

a. Dependent Variable: log10_Sb_Gastric

Gastric bioaccessibility- mine waste

Coefficients[a]

Model		Unstandardized Coefficients B	Unstandardized Coefficients Std. Error	Standardized Coefficients Beta	t	Sig.	Collinearity Statistics Tolerance	Collinearity Statistics VIF
1	(Constant)	-.521	.453		-1.151	.369		
	log10_Sb_Total	.790	.053	.710	14.911	.004	.606	1.650
	log10_Mn	-.588	.439	-.219	-1.341	.312	.051	19.466
	log10_Fe	-.252	.269	-.170	-.936	.448	.042	23.954
	pH	.335	.037	.470	8.986	.012	.501	1.997
2	(Constant)	-.546	.442		-1.234	.305		
	log10_Sb_Total	.812	.046	.730	17.487	<.001	.756	1.323
	log10_Mn	-.984	.112	-.367	-8.816	.003	.760	1.316
	pH	.311	.026	.436	11.897	.001	.979	1.022

a. Dependent Variable: log10_Sb_Gastric

Coefficients[a]

Model		Unstandardized Coefficients B	Unstandardized Coefficients Std. Error	Standardized Coefficients Beta	t	Sig.	Collinearity Statistics Tolerance	Collinearity Statistics VIF
1	(Constant)	-2.301	.622		-3.700	.001		
	log10_Sb_Total	1.209	.269	.709	4.495	<.001	1.000	1.000

a. Dependent Variable: log10_Sb_Gastric

Coefficients[a]

Model		Unstandardized Coefficients B	Std. Error	Standardized Coefficients Beta	t	Sig.	Collinearity Statistics Tolerance	VIF
1	(Constant)	-4.456	2.733		-1.630	.119		
	log10_Sb_Total	3.152	2.413	1.848	1.306	.207	.013	79.050
	logSbTsq	-.386	.477	-1.146	-.810	.428	.013	79.050
2	(Constant)	-2.301	.622		-3.700	.001		
	log10_Sb_Total	1.209	.269	.709	4.495	<.001	1.000	1.000

a. Dependent Variable: log10_Sb_Gastric

Gastric bioaccessibility- dust

Coefficients[a]

Model		Unstandardized Coefficients B	Std. Error	Standardized Coefficients Beta	t	Sig.	Collinearity Statistics Tolerance	VIF
1	(Constant)	-.820	1.180		-.695	.494		
	log10_Sb_Total	1.065	.992	.716	1.073	.294	.026	37.876
	pH	-.131	.167	-.103	-.782	.442	.676	1.480
	LogSbTsq	.030	.221	.094	.137	.892	.025	40.588
2	(Constant)	-.852	1.132		-.753	.459		
	log10_Sb_Total	1.198	.171	.805	7.006	<.001	.852	1.174
	pH	-.141	.146	-.111	-.968	.342	.852	1.174
3	(Constant)	-1.901	.329		-5.786	<.001		
	log10_Sb_Total	1.262	.158	.848	8.003	<.001	1.000	1.000

a. Dependent Variable: log10_Sb_Gastric

Coefficients[a]

Model		Unstandardized Coefficients B	Std. Error	Standardized Coefficients Beta	t	Sig.	Collinearity Statistics Tolerance	VIF
1	(Constant)	-.405	.144		-2.822	.006		
	log10_Sb_Total	-.205	.238	-.187	-.859	.394	.117	8.567
	LogSbTsq	.288	.063	.995	4.578	<.001	.117	8.567
2	(Constant)	-.495	.097		-5.086	<.001		
	LogSbTsq	.237	.021	.819	11.060	<.001	1.000	1.000

a. Dependent Variable: log10_Sb_Gastric

Intestinal bioaccessibility- soil

Coefficients[a]

Model		Unstandardized Coefficients B	Unstandardized Coefficients Std. Error	Standardized Coefficients Beta	t	Sig.	Collinearity Statistics Tolerance	Collinearity Statistics VIF
1	(Constant)	-.917	.699		-1.312	.198		
	log10_Sb_Total	1.120	.126	.828	8.900	<.001	.996	1.004
	pH	-.030	.090	-.031	-.331	.742	.996	1.004
2	(Constant)	-1.126	.302		-3.727	<.001		
	log10_Sb_Total	1.122	.124	.830	9.047	<.001	1.000	1.000

a. Dependent Variable: log10_Sb_Intestinal

Coefficients[a]

Model		Unstandardized Coefficients B	Unstandardized Coefficients Std. Error	Standardized Coefficients Beta	t	Sig.	Collinearity Statistics Tolerance	Collinearity Statistics VIF
1	(Constant)	-.986	.146		-6.746	<.001		
	log10_Sb_Total	1.005	.065	.881	15.494	<.001	1.000	1.000

a. Dependent Variable: log10_Sb_Intestinal

Intestinal bioaccessibility- dust

Coefficients[a]

Model		Unstandardized Coefficients B	Unstandardized Coefficients Std. Error	Standardized Coefficients Beta	t	Sig.	Collinearity Statistics Tolerance	Collinearity Statistics VIF
1	(Constant)	.511	1.331		.384	.708		
	log10_Sb_Total	.774	.545	.368	1.418	.184	.911	1.097
	pH	-.211	.161	-.341	-1.314	.216	.911	1.097
2	(Constant)	-1.112	.512		-2.173	.050		
	log10_Sb_Total	.987	.536	.469	1.840	.091	1.000	1.000

a. Dependent Variable: log10_Sb_Intestinal

Coefficients[a]

Model		Unstandardized Coefficients B	Unstandardized Coefficients Std. Error	Standardized Coefficients Beta	t	Sig.	Collinearity Statistics Tolerance	Collinearity Statistics VIF
1	(Constant)	-1.144	.480		-2.382	.032		
	log10_Sb_Total	1.017	.487	.487	2.086	.056	1.000	1.000

a. Dependent Variable: log10_Sb_Intestinal

Coefficients[a]

Model		Unstandardized Coefficients B	Unstandardized Coefficients Std. Error	Standardized Coefficients Beta	t	Sig.	Collinearity Statistics Tolerance	Collinearity Statistics VIF
1	(Constant)	.344	1.865		.185	.856		
	log10_Sb_Total	-2.408	4.173	-1.153	-.577	.574	.014	71.625
	LogSbTsq	1.852	2.241	1.652	.826	.423	.014	71.625
2	(Constant)	-.720	.273		-2.638	.019		
	LogSbTsq	.568	.258	.507	2.198	.045	1.000	1.000

a. Dependent Variable: log10_Sb_Intestinal

Table S5: Overview of sample digestion methods, bioaccessibility tests, analytical techniques, samples characteristics, reference materials

Reference	Type of sample	Grainsize	Digestion method	BA method	(certified) reference material	analysis
Denys et al. (2009)	kitchen garden soil, slag, soil developed on mined bedrock	<250 µm	?	UBM	Montana soil 2710 and 2711	GFAAS<br>ICP-OES
Sanderson et al. (2012)	Shooting range soils	< 250µm for BA<br>< 2mm for total	Aqua Regia	SBET	BCR 142 — light sandy soil	ICP-OES
Mouton et al. (2014)	Soils from a mining site	Not mentioned	Not mentioned	UBM	IAEA-356 (international atomic energy agency), PACS-2 (national research council of Canada), IPL1 and MIX1 (Pasteur Institute of Lille)	ICP-MS
Baghiferam et al. (2014)	Agricultural field soils	< 2000 µm	Aqua Regia	SBET	no	HG-AAS
Wilson et al. (2014)	Soil samples from floodplain	<250 µm	Aqua Regia, microwave	SBET	NIST SRM 2711 Montana Soil	ICP OES
Li et al. (2014)	Soil samples from a Sb mine and its surroundings	<250 µm	Aqua Regia	SBET and PBET	GBW07408<br>GBW07430	ICP MS
Plumejeaud at al. (2018)	House dust	<150 µm	Aqua Regia	UBM	GRX-1, GRX-4, GRX-6 and SAR-M (United States Geological Survey)<br>BGS 102 bioaccessibility reference material	ICP MS
Hu et al. (2018)	AC filter dust	Not mentioned	Aqua regia	SBRC, PBET	no	ICP-MS<br>ICP OES
Ettler et al. (2018)	Slags and fly ash	Not sieved	digestion in mineral acids ($HClO_4$, HF, HNO_3) and/or alkali sintering	SBRC(G)	SU- 1b (nickel–copper–cobalt ore), certified by CANMET within the framework of the Canadian Certified Reference Materials Project (CCRMP)<br>NIST 1643d (trace elements in water)	ICP OES<br>ICP-MS
Ettler et al. (2019)	Mine tailings, slag dust, smelter dust	< 10 µm	HF, $HClO_4$-HNO_3), Lefort *aqua regia*	SBRC(G)	CCU-1e copper concentrate certified reference material (CCRMP-Canadian Certified Reference Materials project SRM 1640a (Trace elements in natural water) and SRM 1643d (Trace elements in water)	ICP OES<br>ICP-MS
Moreda-Piñeiro et al.(2019)	PM_{10} from industrial, urban and sub-urban sites	< 10 µm	microwave assisted acid digestion with HNO_3 and H_2O_2	PBET	SRM 1648 and ERM-CZ120	ICP-MS
Garau et al. (2019	Soil contaminated by mining	<250 µm	H_2O_2, HNO_3+HCl U.S. EPA Method 3051A), microwave digestion	SBRC	NIST-SRM 2711	ICP MS
Cao et al. (2020)	Soil from e-waste site	<250 µm	HNO_3, HF , H_2O_2 and H_3BO_3, microwave digestion	PBET	NIST 2011a	ICP MS
Ning et al. (2021)	soil from historic Tl\Hg mining area	<180 µm	HNO_3 and HF, hot plate	SBET and PBET	GBW07403 and GBW07408	ICP MS
Diquattro et al. (2021)	Soil spiked with Sb	<250µm	Aqua regia, microwave	SBRC	NIST-SRM 2711a	ICP-QQQ-MS
Amponsah et al. (2022)	Soil from e-waste recycling area	<250 µm	Aqua regia, heating block	Method 1340	NIST 2711	ICP MS

Hiller et al (2022)	Soils from urban community gardens	<250µm	$HClO_4$–HF digestion, hot plate	PBET	NIST 2711a – Montana II soil	ICP MS
Wang et al. (2023)	Soil around abandoned ordnance plant	<150µm	HCl and HNO_3 in a Teflon bomb, digested in a hot water bath	SBET	Not mentioned	AFS
Kastury et al. (2024)	legacy gold mining waste	<250 µm	Aqua regia, microwave	Method 1340	NIST 2710a	ICP OES
Gabersek and Gosar (2025)	soil, attic dust, street dust, household dust	<250 µm	HF-HNO_3-$HClO_4$ digestion	UBM	2711a, BCR-2, BGS-102, NIST 2584	ICP MS
